# Protective Efficacy of Centralized and Polyvalent Envelope Immunogens in an Attenuated Equine Lentivirus Vaccine

**DOI:** 10.1371/journal.ppat.1004610

**Published:** 2015-01-08

**Authors:** Jodi K. Craigo, Corin Ezzelarab, Sheila J. Cook, Chong Liu, David Horohov, Charles J. Issel, Ronald C. Montelaro

**Affiliations:** 1 Center for Vaccine Research, University of Pittsburgh, Pittsburgh, Pennsylvania, United States of America; 2 Department of Microbiology and Molecular Genetics, University of Pittsburgh, Pittsburgh, Pennsylvania, United States of America; 3 Department of Veterinary Science, Gluck Equine Research Center, University of Kentucky, Lexington, Kentucky, United States of America; University of North Carolina at Chapel Hill, United States of America

## Abstract

Lentiviral Envelope (Env) antigenic variation and related immune evasion present major hurdles to effective vaccine development. Centralized Env immunogens that minimize the genetic distance between vaccine proteins and circulating viral isolates are an area of increasing study in HIV vaccinology. To date, the efficacy of centralized immunogens has not been evaluated in the context of an animal model that could provide both immunogenicity and protective efficacy data. We previously reported on a live-attenuated (attenuated) equine infectious anemia (EIAV) virus vaccine, which provides 100% protection from disease after virulent, homologous, virus challenge. Further, protective efficacy demonstrated a significant, inverse, linear relationship between EIAV Env divergence and protection from disease when vaccinates were challenged with viral strains of increasing Env divergence from the vaccine strain Env. Here, we sought to comprehensively examine the protective efficacy of centralized immunogens in our attenuated vaccine platform. We developed, constructed, and extensively tested a consensus Env, which in a virulent proviral backbone generated a fully replication-competent pathogenic virus, and compared this consensus Env to an ancestral Env in our attenuated proviral backbone. A polyvalent attenuated vaccine was established for comparison to the centralized vaccines. Additionally, an engineered quasispecies challenge model was created for rigorous assessment of protective efficacy. Twenty-four EIAV-naïve animals were vaccinated and challenged along with six-control animals six months post-second inoculation. Pre-challenge data indicated the consensus Env was more broadly immunogenic than the Env of the other attenuated vaccines. However, challenge data demonstrated a significant increase in protective efficacy of the polyvalent vaccine. These findings reveal, for the first time, a consensus Env immunogen that generated a fully-functional, replication-competent lentivirus, which when experimentally evaluated, demonstrated broader immunogenicity that does not equate to higher protective efficacy.

## Author Summary

Our best effort for containment of the global HIV epidemic is the development of a broadly protective vaccine. Current research has focused on vaccines that can generate a protective immune response against numerous strains of the virus. For this reason, vaccines with centralized HIV genes as immunogens, which merge HIV genetic information and potentially protect against multiple viral strains in a single inoculation, are an increasing area of interest to the field. Existing published studies have not evaluated centralized immunogens in the context of attenuated vaccines, which to date, have demonstrated the highest level of vaccine protection in lentiviral studies. Furthermore, centralized immunogen studies have also not included protective efficacy findings accomplished through challenge with highly pathogenic virus strains. In this study we not only examine the immunogenicity of these immunogens in an animal model, but we also for the first time evaluate the ability of centralized immunogens to induce protection against virulent virus challenge.

## Introduction

The scientific community has aggressively sought after the development of a universal HIV vaccine that can prevail over the extraordinary levels of antigenic diversity in the fight against HIV and AIDS. The considerable extent of genomic variation found between isolates and within clades, and to a larger extent within the circulating recombinant forms, make for an effectual blockade to vaccine protection. Different strategies of vaccine composition and delivery have been proposed that are actively and widely being examined. A majority of these vaccines target the Env protein, as lentiviral antigenic variation is most pronounced in the viral Env proteins that serve as initial primary targets for host immune responses [1]–[5].

Centralized Env immunogens are one of the more promising contemporary approaches to overcoming HIV antigenic diversity [1], [6]. Centralized sequences attempt to minimize the genetic distance between vaccine proteins and the circulating isolates that pose a threat to public health. The centralized genes are generated through the computational determination of consensus genes (the most common amino acid at each position), ancestral genes (modelling ancestral states through phylogenetics), or center of the tree sequences (phylogenetic determination of a central isolates) [1], [4], [7], [8]. Centralized genes have been investigated as effective vaccine approaches in the HIV field both as DNA and/or protein immunogens [6], [9]–[19]. To date, however, the efficacy of centralized immunogens has not been fully explored in the context of an attenuated lentiviral vaccine model that could provide both immunogenicity data as well as protective efficacy data via virulent challenge in an animal model.

Equine infectious anemia virus (EIAV), a macrophage-tropic lentivirus, causes a persistent infection and chronic disease in equids [20]. Infection, transmitted via blood-feeding horse flies, occurs in three stages: acute, chronic and inapparent. Acute and chronic stages are defined by episodes of clinical disease that are triggered by waves of viremia, and distinguished by fever, anemia, thrombocytopenia, edema, and various wasting signs. By one year post-infection animals typically progress to life-long inapparent carriers, but continue to harbor steady-state levels of viral replication in monocyte-rich tissue reservoirs [20]–[22]. Stress or immune suppression of inapparent carriers can induce increases in viral replication and potentially a recrudescence of disease [20], [23]. Among virulent lentiviruses, EIAV is unique in that, despite aggressive virus replication and associated rapid antigenic variation, greater than 90% of infected animals progress from chronic disease to an inapparent carrier stage, by a strict immunologic control over virus replication [20]. The EIAV system hence serves as a unique animal model for the natural immunologic control of lentiviral replication and disease. Further, EIAV inapparent carriers have proven to be resistant to subsequent virus exposure to diverse viral strains, indicating the development of a high level of prophylactic immunity. Thus, the EIAV system provides a valuable model for identifying critical immune correlates of protection and ascertaining the potential for developing effective prophylactic lentivirus vaccines [24].

While the disease processes for EIAV and HIV have distinguishing dynamics, key similarities between the two virus systems make EIAV an extremely valuable tool and model for AIDS vaccine development [24]–[26]. EIAV and HIV are transmitted parenterally and share a macrophage/monocyte tropism [26], [27]. EIAV quasispecies also possess high levels of antigenic heterogeneity and their Env proteins share architectural characteristics such as extensive glycosylation and immune decoys [24], [25], [28], [29]. These features, all of which are critical elements associated with initial virus exposure, coupled to a very similar immune maturation process of the EIAV-infected equine to HIV-infected humans, are fundamental factors relevant to vaccine efficacy [24], [30].

We previously reported serial studies evaluating the efficacy of an attenuated EIAV proviral vaccine containing a mutation in the viral *S2* accessory gene (EIAV_D9_) [31]–[33]. The results of these studies indicate that horses inoculated with the EIAV_D9_ viral vaccine were 100% protected from disease by virulent, albeit homologous, EIAV challenge. Thus, the EIAV system mirrors other animal lentivirus vaccine models, which consistently identify attenuated vaccines as producing the highest level of vaccine protection. [31]–[38]. Our latest EIAV_D9_ data demonstrated the effects of challenge virus Env sequence variation on vaccine protection [39]–[41]. We identified a significant, inverse, linear correlation between vaccine efficacy and increasing divergence of the challenge virus Env surface protein, gp90, compared to the vaccine virus gp90 protein. The study demonstrated that the 100% protection of immunized horses from disease after challenge by virus with a homologous gp90 (EV0), dropped to approximately 60% protection when a challenge virus gp90 was 6% divergent (EV6), and nose-dived to less than 50% protection against challenge with a gp90 that was 13% (EV13) divergent from the vaccine strain. Most recently, we demonstrated that the attenuated vaccine strain progressively evolved during the seven-month pre-challenge period and that the observed protection from disease was significantly associated with divergence from the original vaccine strain, not the overall diversity of the vaccine Env quasispecies present on the day of challenge (DOC) [39].

Despite numerous studies on the immunogenicity of centralized Env proteins, use of these noteworthy immunogens in an attenuated vaccine model, accompanied by virulent virus challenge, has yet to be reported. In the current study, we sought to directly build upon our current model and the series of described EIAV_D9_ vaccine studies. Our attenuated vaccine model, coupled with the well-characterized genomic and phylogenetic ancestry of the Env gene of our EIAV strain, enabled a thorough, unparalleled evaluation of centralized sequence vaccine efficacy not as readily modelled in other lentiviral systems. The presented studies evaluated multiple derivatives of centralized Env immunogens, both consensus and ancestral, in our proviral attenuated vaccine backbone. The studies were designed to first, develop and test a consensus immunogen for functionality through examination of replication and pathogenic potential in proviral backbones; second, compare the protective efficacy of the consensus immunogen in an attenuated backbone to attenuated strains containing our ancestral Env [34] as well as a polyvalent Env attenuated strain mixture; and third, to develop and utilize a more stringent challenge model in the form of an engineered quasispecies.

## Results

### Development of consensus Env

**Figure 1:**
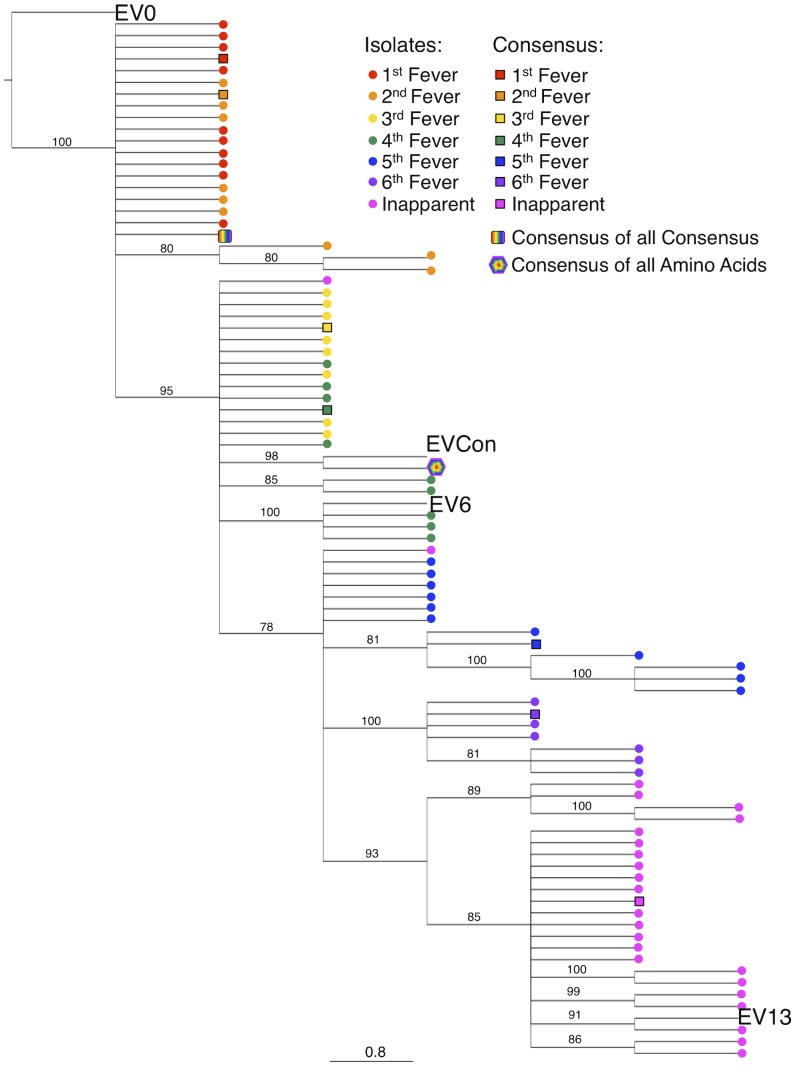
Phylogenetic analyses of the ConEnv, all consensus Envs, and the longitudinal isolates derived from experimental infection utilized in consensus gene development. A phylogenetic tree of aligned plasma amino acid sequences (gp90 regions) was constructed by the neighbor joining method from Kimura corrected distances with the optimality criterion set to distance. The tree was rooted to the ancestral Env, EV0. Bootstrap values were determined over 1000 iterations and are indicated at the nodes of the branches. Branch lengths are proportional to the distance existing between the sequences.

**Figure 2:**
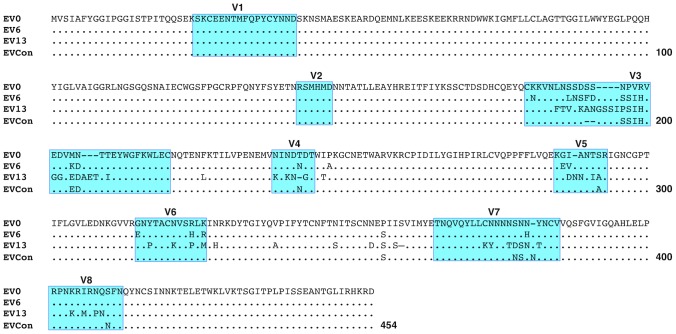
Comparison of deduced amino acid gp90 variable region sequences from EIAV variant Envs (used in attenuated and challenge strain construction) and ConEnv. The region of *env* gene coding for the surface glycoprotein, gp90, of the Env variants, EV0, EV6, and EV13 (ref) was compared to the ConEnv sequence. Deduced amino acid sequences were aligned and compared to EV0 (ancestral Env that is analogous to the EIAV_D9_ Env). Only the amino acid residues different from EV0 are reported on the alignment. Dots (·) indicate residues identical to the EV0 sequence; dashes (-) indicate amino acid deletion; Previously described variable regions V1 through V8 are boxed and shaded in blue.

Consensus gene development of the EIAV Env protein focused on the gp90 region of the gene as genomic evolution and antigenic variation in the transmembrane (gp45) protein has been shown to be minimal among characterized longitudinal EIAV isolates [42], [43]. To engineer a consensus Env, the gp90 genes of approximately 90 naturally occurring isolates from an experimental infection [42], [44] were aligned. Virus isolates included the inoculum strain Env as well as isolates from all three stages of disease (acute, chronic, and inapparent). Isolates therefore included an ancestral strain (EV0) and its descendant strains that evolved innately between day zero and 1200 days post-infection (DPI). Consensus sequences were derived primarily from codon-aligned nucleotide sequences and secondarily from amino acid alignments. The consensus sequence from the nucleotide alignment was translated, compared to the consensus sequence from the amino acid alignment for congruence and the resolution of ambiguities, and termed ConEnv. To evaluate the veracity of the derived ConEnv sequence and discriminate it against other potential consensus Env proteins, additional consensus sequences were designed. Consensus Envs representing the individual febrile episodes (six) and inapparent stage isolates were generated from the isolate amino acid sequences and thereafter a consensus from those engineered sequences was constructed as well. This “consensus from all consensus” method is similar to creating consensus Envs from each HIV clade and subsequently creating a consensus of those clades. The ConEnv consensus sequence was then examined by phylogenetic comparison with these control consensus sequences, the Env sequences involved in ConEnv construction, and sequences targeted as partner Envs for vaccine and challenge strains of this study (Fig. 1). The “consensus from all consensus” sequence was phylogenetically closely related to the ancestral Env (EV0) emerging on the main ancestral root amongst the acute disease Env genes. ConEnv shared the same ancestral root as the EV6 strain, manifesting ancestrally between EV0 and EV6. Genetic distance calculations demonstrated the ConEnv gp90 sequence was 4%, 6%, and 11% divergent from the EV0, EV6, and EV13 strains, respectively. The majority of the disparate residues occurred within the designated variable regions, specifically V3 through V7 (Fig. 2). Hence, the ConEnv sequence was a strong consensus sequence representative of the 90 isolates, capturing epitopes broadly characteristic of the family of EIAV isolates.

### Functional replication assessment and characterization of the consensus Env

To fully assess the competency of the ConEnv protein to function indistinguishably from a naturally occurring Env protein, ConEnv was evaluated in the context of both attenuated and pathogenic EIAV proviruses. Commercially synthesized ConEnv was cloned into the attenuated EIAV_D9_ backbone [31], [32] and the pathogenic EIAV_UK3_ backbone [45], with the resultant proviral strains termed ConD9, and EVCon, respectively. Attenuated and pathogenic proviral clones were sequenced to verify the ConEnv gene, and then transfected into equine dermal (ED) cells for production of infectious virus stocks. Virus stocks were titered and characterized for *in vitro* replication kinetics [46]–[48]. Both proviral strains, EVCon (pathogenic) and ConD9 (attenuated) demonstrated typical *in vitro* kinetics, emulating their parental and variant strain counterparts, and peaked in virus production at approximately ten DPI.

**Figure 3:**
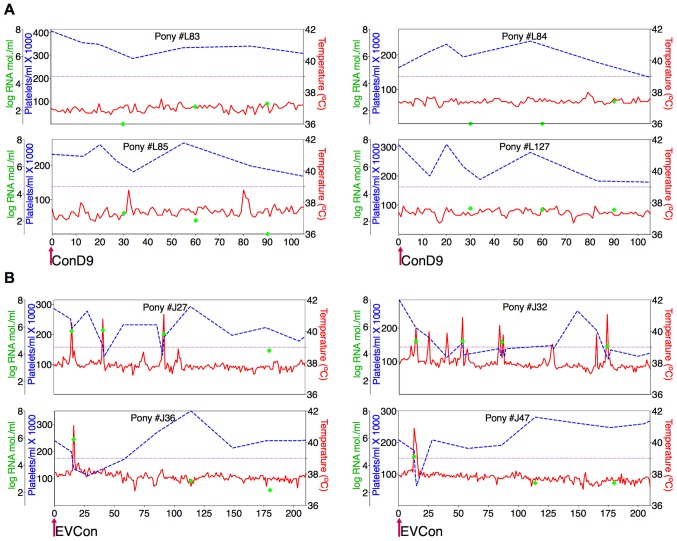
Clinical and virological profiles of ponies inoculated with the consensus Env in attenuated (ConD9) and pathogenic (EVCon) EIAV viral backbones. EIAV-naïve ponies (8 total) were inoculated intramuscularly with 1×10^3^ TCID_50_ (pink arrow) of EIAV strains containing the consensus Env in either an (**A**) attenuated backbone, ConD9, or in (**B**) our pathogenic backbone, EVCon. Rectal temperature (red line, right Y axis) and platelet counts (blue dashed line, first left Y axis) were followed daily (X-axis). Quantification of the virus load (green diamond, second left Y axis) was performed on viral RNA extracted from plasma at periodic time points. Febrile episodes were defined by a combination of two-three features, including: rectal temperature above 39°C in conjunction with thrombocytopenia (platelet decrease of ≥70,000/µl of whole blood), EIAV viral load ≥10^5^ as well as other clinical signs of EIA.

*In vivo* analysis of the proviral pathogenic and attenuated ConEnv strains, by experimental infections of equids, confirmed characteristic EIAV clinical and virological profiles of both pathogenic and attenuated infections (Fig. 3). Typical attenuated and avirulent replication properties were observed for the ConD9 strain. Low level, viral replication kinetics (averaging between 10^2^–10^3^ copies RNA/ml) which failed to progress to clinical disease over a 100 day observation period were observed. Conversely, pathogenesis and virulence, characterized by standard viremic replication kinetics (averaging between10^3^–10^4^ copies RNA/ml and peaking at 10^6^ copies RNA/ml), including the induction of acute, and progression to chronic disease were observed with the EVCon strain [20], [43], [49]. Hence, for the first time, a synthetic consensus lentiviral Env was demonstrated to not only be fully functional in the context of replication competence *in vitro* and *in vivo*, but also capable of inducing traditional and virulent lentiviral disease.

**Figure 4:**
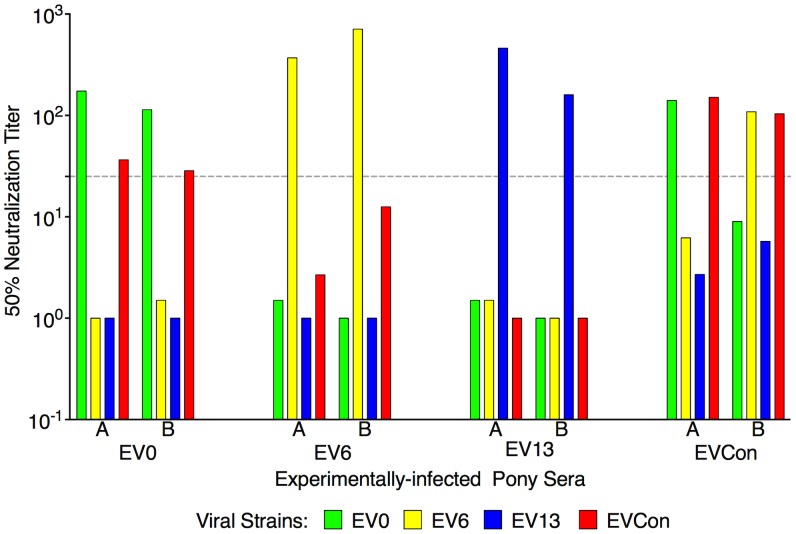
Neutralization profiles of proviral variant strains with serum from animals experimentally infected with EV0, EV6, EV13, or EVCon. Variant proviral strains were tested for *in vivo* pathogenecity through experimental infections of ponies. Serum from the infections was utilized here to test for immunogenicity and characterize the variant Envs. At six-months post-infection serum from two representative animals (labeled A,B) was tested in our standard neutralization assay. The mean reciprocal dilutions of serum which neutralized 50% of input EV0, EV6, EV13, or EVCon as measured in an infectious center assay are presented. The dashed line denotes the cut off (≥25) value for valid 50% neutralization titers.

The final pre-vaccine trial evaluation was an assessment of ConEnv's immunogenicity. Assays of antibody responses elicited by EVCon, EV0, EV6, and EV13 experimental infections, with variant and consensus viruses, indicated primarily distinct neutralization phenotypes for the individual variant Envs; each variant virus was neutralized by immune serum from homologous virus infections, but not from heterologous virus infections, except for marginal neutralization of the EVCon strain by the EV0 heterologous serum (Fig. 4). However, sera produced by the EVCon virus infections were capable of not only neutralizing its homologous strain, but also neutralized the EV0 and EV6 heterologous strains. Thus, these data demonstrate that the ConEnv was similar to EV0, EV6, and EV13 in replication and virulence properties [34], yet distinct in immune properties as a result of the defined Env sequence variation. Much like the HIV consensus Env recombinant proteins that have been reported [6], [15]–[18], this consensus Env, in the context of a fully functional virus, demonstrated immunogenicity induction of neutralizing antibodies with broader recognition of epitopes than that of the naturally occurring isolates from which the ConEnv was derived.

### Experimental vaccination and challenge

To directly evaluate the consensus Env as well as the general premise of centralized immunogens, we compared the consensus Env attenuated strain (ConD9) with an ancestral Env attenuated vaccine strain (EIAV_D9_ or D9). Proficiency of the centralized immunogens was further scrutinized by inclusion of a third attenuated vaccine regimen. The third arm of the study, a polyvalent attenuated strain mixture, was chosen as the most rigorous match to the centralized immunogens. The polyvalent attenuated quasispecies was constructed utilizing the D9 backbone. A trivalent attenuated mixture was assembled with the D9 as one of three strains. The EV6 and EV13 Envs were cloned into the D9 backbone to create 6D9 and 13D9, respectively, the final two strains of the polyvalent mix. The polyvalent attenuated mixture, a 1∶1∶1 mix of D9, 6D9, and 13D9, otherwise termed TriD9, was tested *in vivo* for TCID_50_ dosage verification in a group of eight ponies.

**Figure 5:**
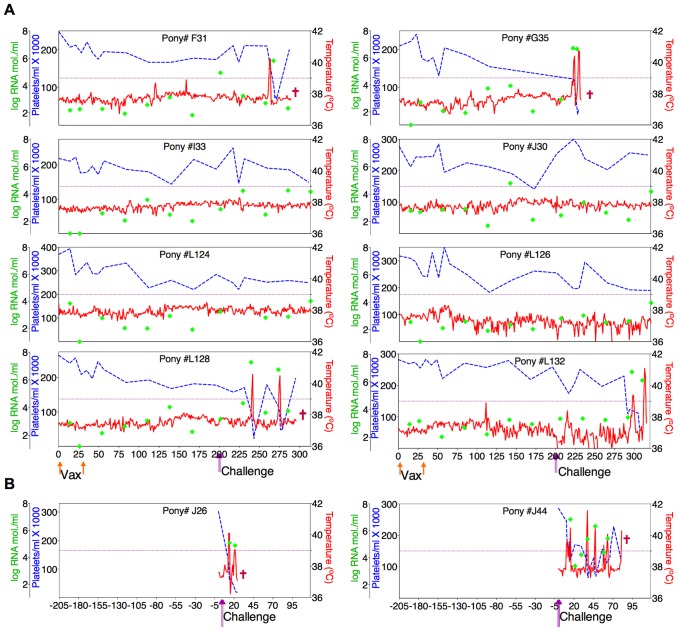
Clinical and virological profiles of D9 vaccine trial group. (**A**) Eight EIAV-naïve ponies were vaccinated with 3×10^3^ TCID_50_ D9 I.M. (orange arrow, Vax). Rectal temperature (red line, right Y axis) and platelet counts (blue dashed line, first left Y axis) were followed daily for up to 320 days (X-axis) after the first vaccine dose. Quantification of the virus load (green diamond, second left Y axis) was performed on viral RNA extracted from plasma at periodic time points prior to and after virulent virus challenge seven months post-first vaccination with 3×10^3^ TCID_50_ EV_MX_ (1∶1∶1 - EV0, EV6, or EV13) I.M (pink arrows, Challenge). (**B**) Two EIAV-naïve ponies were also challenged with 3×10^3^ TCID_50_ EV_MX_ (1∶1∶1 - EV0, EV6, or EV13) I.M (pink arrows, Challenge). Febrile episodes were defined by a achieving a combination of two-three features such as: rectal temperature above 39°C in conjunction with thrombocytopenia (platelet decrease of ≥70,000/µl of whole blood), EIAV viral load ≥10^5^ as well as other clinical signs of EIA. Burgundy cross symbol, animal euthanized due to severe disease or as endpoint of study.

**Figure 6:**
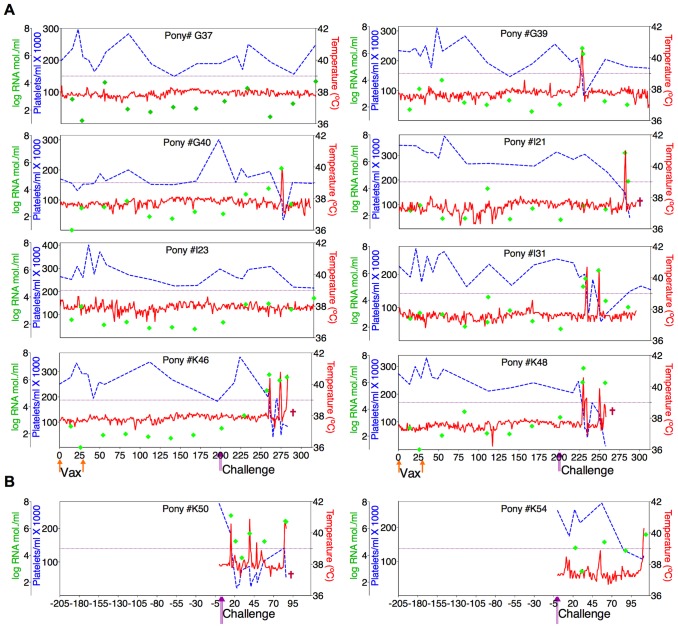
Clinical and virological profiles of ConD9 vaccine trial group. (**A**) Eight EIAV-naïve ponies were vaccinated with 3×10^3^ TCID_50_ ConD9 I.M. (orange arrow, Vax). Rectal temperature (red line, right Y axis) and platelet counts (blue dashed line, first left Y axis) were followed daily for up to 320 days (X-axis) after the first vaccine dose. Quantification of the virus load (green diamond, second left Y axis) was performed on viral RNA extracted from plasma at periodic time points prior to and after virulent virus challenge seven months post-first vaccination with 3×10^3^ TCID_50_ EV_MX_ (1∶1∶1 - EV0, EV6, or EV13) I.M (pink arrows, Challenge). (**B**) Two EIAV-naïve ponies were also challenged with 3×10^3^ TCID_50_ EV_MX_ (1∶1∶1 - EV0, EV6, or EV13) I.M (pink arrows, Challenge). Febrile episodes were defined by a achieving a combination of two-three features such as: rectal temperature above 39°C in conjunction with thrombocytopenia (platelet decrease of ≥70,000/µl of whole blood), EIAV viral load ≥10^5^ as well as other clinical signs of EIA. Burgundy cross symbol, animal euthanized due to severe disease or as endpoint of study.

**Figure 7:**
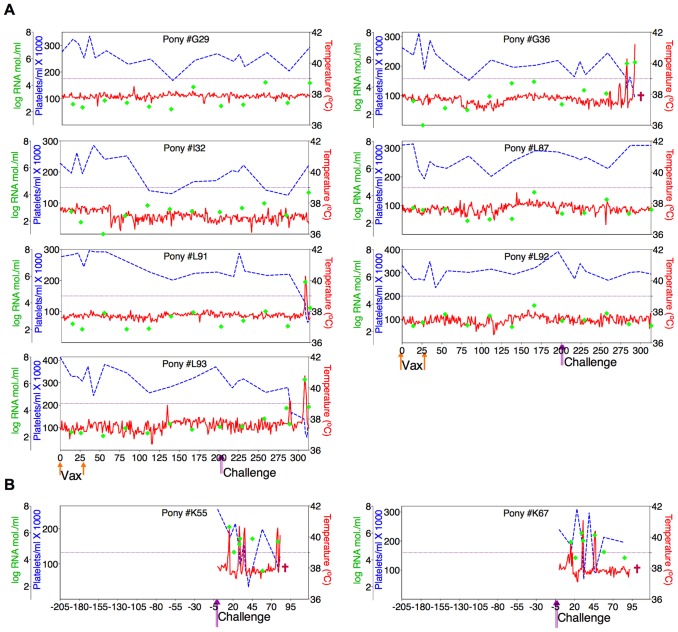
Clinical and virological profiles of TriD9 vaccine trial group. (**A**) Seven of eight EIAV-naïve ponies vaccinated with 3×10^3^ TCID_50_ TriD9 I.M. (orange arrow, Vax) are presented. One of the eight original vaccinates experienced clinical complications and was removed from the trial. Rectal temperature (red line, right Y axis) and platelet counts (blue dashed line, first left Y axis) were followed daily for up to 320 days (X-axis) after the first vaccine dose. Quantification of the virus load (green diamond, second left Y axis) was performed on viral RNA extracted from plasma at periodic time points prior to and after virulent virus challenge seven months post-first vaccination with 3×10^3^ TCID_50_ EV_MX_ (1∶1∶1 - EV0, EV6, or EV13) I.M (pink arrows, Challenge). (**B**) Two EIAV-naïve ponies were also challenged with 3×10^3^ TCID_50_ EV_MX_ (1∶1∶1 - EV0, EV6, or EV13) I.M (pink arrows, Challenge). Febrile episodes were defined by a achieving a combination of two-three features such as: rectal temperature above 39°C in conjunction with thrombocytopenia (platelet decrease of ≥70,000/µl of whole blood), EIAV viral load ≥10^5^ as well as other clinical signs of EIA. Burgundy cross symbol, animal euthanized due to severe disease or as endpoint of study.

Twenty-four ponies of mixed age and gender were divided randomly into groups of eight animals and inoculated intravenously with two, 3×10^3^ TCID_50_ doses of the ancestral D9 vaccine, the consensus ConD9 or polyvalent TriD9 vaccines, at four-week intervals. The inoculated ponies were monitored daily for clinical signs of EIA, and blood samples were taken at regular intervals for standard measurements of disease, virus replication, and host immune responses, as described previously [31]–[33], [50]. Figs. 5–7 display the clinical profiles of vaccinated animals. One of the eight animals in the polyvalent TriD9 group developed clinical complications pre-challenge that compromised continued use of the animal in the study and was thus removed from the trial. All twenty-three vaccinates (3 trial groups) exhibited no clinical signs of EIA disease from the attenuated vaccine strains during the seven month observation period, a time frame that allows complete maturation of vaccine immunity prior to virus challenge [22], [30], [51] (Figs. 5A–7A).

An engineered quasispecies challenge model, EV_MX_, was developed as a rigorous assessment of immune protection. Based on previous studies [28], [34], equivalent (1∶1∶1) TCID_50_ dosages of the virulent EV0, EV6, and EV13 strains combined to create the virulent, well-defined, swarm. Six months following the second vaccination, the immunized ponies were challenged intravenously every other day with three, 3×10^3^ TCID_50_, EV_MX_ inoculations. A control group consisting of six EIAV-naïve ponies was also challenged with EV_MX_ (Figs. 5B–7B). Analyses of vaccinate day of challenge (DOC) viral loads demonstrated all three attenuated viral regimens replicated to similar levels, averaging, over the seven month pre-challenge period, between 2×10^3^ and 3×10^3^ copies RNA/ml plasma (Figs. 5–7). Despite these similarities in viral vaccine replication, however, trial groups displayed markedly different levels of EV_MX_-induced disease. Four ancestral D9, six consensus ConD9, and three polyvalent TriD9 animals displayed clinical signs of EIA disease during the observation period post challenge (Figs. 5A–7A). Chronic disease was observed in the majority of vaccinates that experienced initial acute disease. All six control animals of each variant virus challenge group developed clinical EIA disease, indicating 100% virulence of the quasispecies challenge under the current experimental infection conditions. DOC vaccine immune responses in all groups were also indicative of mature immune responses (Table 1, Table 2, S1 Fig.). The polyvalent TriD9 group demonstrated the highest Env-specific serum antibody titer and avidity, although it was only significantly different from the ancestral D9 regimen (*P* = 0.017, *P*<0.0001, respectively), and not from the consensus ConD9 regimen (S1 Fig.). While neutralizing antibodies were detectable in all three groups, they could not be associated with protection. Similarly, DOC cellular immunity levels were similar but not correlated with protection levels (Table 2). For example, measured *in vivo* cytokine responses were the lowest in the polyvalent TriD9 vaccinates that showed the highest level of protection.

**Table 1:** Characterization of vaccine Env-specific antibody prior to challenge.*

Trial Group	Reciprocal Antibody Titer [10^3^]				Avidity^a^	Conformation^b^
	PV (EV0, D9)	EVCon	EV6	EV13	PV (EV0, D9)	PV (EV0, D9)
D9	0.62 (±0.45)	-	-	-	17 (8)	1.3 (0.37)
ConD9	2.5 (±2.7)	1.34 (±0.56)	-	-	33 (12)	1.45 (0.31)
TriD9	3.77 (±4.1)	-	1.86 (±0.62)	1.66 (±0.39)	37 (9)	1.12 (0.22)

*Numbers represent averages for each trial group, All values in () are SD.

a Avidity determines the relative resistance of serum antibody;antigen binding to washes with 8M urea in ELISA assays, as described (22).

b Conformation ratios reflect the relative serum antibody reactivity to linear or conformational epitopes by comparisons of antibody reactivity with intact and denatured Env antigens in ELISAs, as described (22); Conformation ratio = denatured Env reactivity/native Env reactivity.

**Table 2:** Characterization of vaccine immune responses prior to challenge.*

Trial Group	Reciprocal Neutralizing Antibody Titer^a^			gp90-Specific Cellular Immune Responses, RQ^b^				
	PV/EV0	EVCon	EV_MX_	INFγ	GrzB	CD8	CD4	IL-2
D9	97 (±59)	-	-	76 (±20)	3 (±0.3)	8 (±2)	182 (±116)	4 (±1)
ConD9	38 (±7)	<25	-	212 (±62)	3 (±0.4)	4 (±1)	7 (±2)	6 (±2)
TriD9	32 (±13)	-	<25	6 (±2)	1 (±0.1)	2 (±1)	2 (±0.4)	1 (±0.4)

*Numbers represent averages for each trial group, All values in () are SD.

a Serum Neutralization titer analyses were against EIAV_PV_. Positive neutralization determined as reciprocal titers >25 (27, 40).

b Pooled responses of all vaccinate-specific peptides; RQ, Relative Quantity, determined as outlined in Materials and Methods.

INFγ, Interferon-γ; GrzB, Granzyme B.

**Figure 8:**
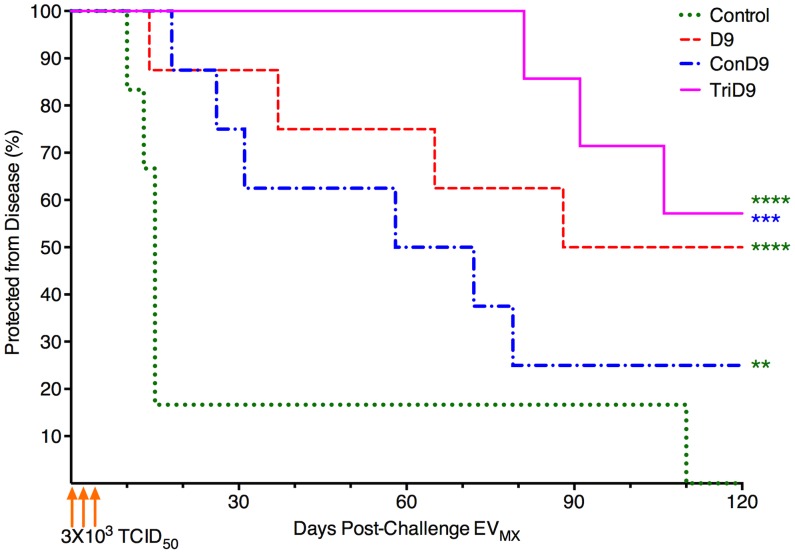
Protection from disease is associated with complexity of immunogen. The percentage of animals within each trial group protected from clinical EIA was plotted as a function of days post-challenge with the engineered quasispecies EV_MX_. Overall resulting protection curves are significantly different from one another (ANOVA, *P*<0.0001). TriD9 demonstrated the highest level of protection (****), followed by D9 (****). The consensus Env, or ConD9 attenuated vaccine, conferred the lowest level of protection; however, it was significant as compared to unvaccinated subjects (**). The TriD9 vaccinated animals were also significantly more protected than the ConD9 animals (***). Logrank test for trend via survival curve analysis reveal a significant trend for the complexity of the immunogen and protective efficacy (*P* = 0.02.) Multiple comparisons performed via Bonferroni's multiple comparison test: ****, *P*<0.0001; ***, *P* = 0.0002, **, *P* = 0.001.

The percentage of animals within each trial group protected from clinical EIA was plotted as a function of days post-challenge for survival analysis (Fig. 8). Both centralized Env vaccine groups had subjects that succumbed to EV_MX_ disease within a typical time frame of 2–3 weeks post-challenge. The polyvalent, TriD9 group, however, demonstrated a delay in the onset of disease with the first animal not breaking until 81 days post-challenge (Fig. 8). Overall, protection curves of all three vaccine groups were significantly different from one another (ANOVA, *P*<0.0001). Polyvalent TriD9 vaccinates demonstrated the highest levels of protection that was significantly different from the unvaccinated controls and the consensus ConD9 curves (*P*<0.0001, *P* = 0.0002). While the consensus ConD9 group had the lowest level of protection, it was significant as compared to unvaccinated subjects (*P* = 0.001). Analysis of the trend revealed a significant relationship between the complexity of immunogen and protective efficacy (*P* = 0.02). Ultimately, the consensus ConD9 strain, while pre-trial appearing to be more broadly immunogenic, demonstrated the lowest level of protection. The polyvalent TriD9 regimen demonstrated the highest level of protection against a quasispecies challenge. The current study not only highlighted important information towards HIV vaccine development and highlighted the importance of rigorous challenge strain engineering.

## Discussion

The collective success of attenuated vaccine regimens makes it the ideal modality to rigorously test novel vaccine concepts. Previous work by our group and others have demonstrated attenuated vaccines to be an extremely useful tool for vaccine development, regardless of the potential for marketable advancement of the attenuated platform due to the safety concerns associated with HIV vaccines. Consequently, we resolved to examine the efficacy of centralized Env immunogens in our well-established EIAV attenuated vaccine model. Results presented here reveal, for the first time, a consensus Env in a fully replication-competent attenuated virus backbone that can confer protective efficacy against virulent virus challenge; however, it does not induce the highest level of protection as compared to ancestral or polyvalent vaccines.

A concept not articulated in most reports on centralized Env sequences are the specifics involved in the determination of the consensus sequence. Creation of an ideal consensus sequence requires careful consideration of various parameters. Our original attempts to construct a consensus gp90 gene focused on a more basic approach than what we utilized for the current study. Initial alignments were performed utilizing the three sequences that comprise the EV_MX_ quasispecies challenge strain, EV0, EV6, and EV13 [34]. Phylogenetic analysis of this consensus gp90 demonstrated that the consensus sequence was located high on the ancestral root and would not have represented a good consensus sequence as it was highly related to the ancestral, or EV0 gp90. Likewise, the “consensus of all consensus” method utilized in both the HIV and influenza fields [6], [9], [15], [18], [19], [52]–[56] resulted in a gp90 sequence that phylogenetically was more related to early disease isolates on the ancestral root (Fig. 1) than to the consensus gene generated from all 90 isolate sequences (nucleotide and amino acid). *In* vitro analysis of the virus constructed from the second-generation consensus gp90, assembled from the 90-isolate alignment and cloned into a proviral backbone, was found to infect equine cells, but was not fully replication competent. Therefore, if we were to analyze this Env in a single-round infection assay it would falsely appear to be fully functional. Hence, the 90-isolate sequence alignment was re-evaluated and a higher level of hand-editing performed, especially in the highly variable V3 region, prior to consensus generation. This thorough computational analysis and consideration of the consensus Env limited the potential sampling bias that obfuscates computational engineering of protein immunogens [1], [57]. Likewise, our use of an actual ancestoral Env also reduced the potential sampling bias that is problematic to computationally constructed most recent common ancestors [1], [57]. Additionally, in the absence of replication analysis and study in an attenuated model, the incapability of the consensus Env to functional naturally would not have been observed and a gp90 less representative of a fully functional Env would have been examined. Ultimately, this is the first successful construction of a consensus envelope lentivirus construct with full replication and virulence properties.

The highest protective efficacy against disease was observed in the polyvalent TriD9 vaccinates. Survival analysis revealed the polyvalent TriD9 disease curve was significantly different from the naïve controls and importantly, the consensus ConD9 curve. Considering the EV_MX_ challenge quasispecies virus strain composition, the polyvalent TriD9 regimen displaying the highest levels of protective efficacy might be anticipated; however, the ancestral D9 vaccinates also protected to a higher degree than the consensus ConD9 vaccinates. Pre-challenge analysis of clinical and virological factors would not have predicted these results. All three attenuated vaccine regimens displayed similar levels of pre-challenge viral strain replication. Pre-trial evaluation of the capability to induce neutralizing antibodies (Fig. 4) indicated that the ConEnv produced a broader immune response than the ancestral gp90 (EV0/D9) and also the EV6 and EV13 gp90 proteins. Immune analysis of DOC responses did not reveal a direct correlation of protection. Neutralizing antibodies and cellular immune responses were not associated with protective efficacy. Although a significantly higher level of Env-specific antibodies were found in the polyvalent TriD9 vaccinates, the consensus ConD9 vaccinates, similarly, had a higher antibody response as compared to the ancestral D9. In the case of the consensus ConD9 vaccinates, immune response data is suggestive of potential issues related to the artificial nature of the consensus Env and its ability to induce broadly protective responses. Protection in all three vaccine groups could be due to anamnestic responses that could be related to conserved regions or conformational epitopes that allow for protective response in lieu of sequence identity. Although total antibody binding does not reveal directly correlative data (S1 Fig.), mapping of the reactive epitopes will be key to determining if a region of the gp90 potentially conferred more protective responses. These studies are currently underway. A notable observation of the polyvalent TriD9 vaccinates was the delayed onset of disease (Fig. 8). The quasispecies challenge virus eventually broke through causing its first case of disease at the late time point of 81 days post-challenge. The nature of this break through is an interesting study of evolution: did the EV_MX_ Env evolve at a faster rate than the polyvalent TriD9 and escape the protective immune responses or did recombination allow the escape. Current studies are being performed to enable future characterization of break-through febrile isolates.

As part of the current report we generated an engineered quasispecies challenge virus mix containing different degrees of Env variation. The majority of current lentiviral vaccine studies employ a single strain challenge model. Heterologous challenge models, while more rigorous than homologous challenge models, are commonly single viral strains. Data presented here indicate that more comprehensive challenge models, that include variable Env proteins, should be developed for the study of lentiviral vaccines to better test the vaccine modalities being investigated. The polyvalent TriD9 and EV_MX_ vaccine and challenge strains were at their basic phenotype, a homologous pairing. However, the complexity of a pathogenic, diverse, challenge strain resulted in a notable difference and reduction in the protective efficacy as compared to previous studies where the vaccine and challenge strains were matched in their Env sequences. Improved challenge model development for animal lentiviral studies that comprehensively test protective efficacy are critical tools required for broadly protective vaccine development.

The studies presented here demonstrate definitively that polyvalent attenuated vaccine regimens have significantly higher levels of protection as compared to centralized immunogens. Although it is not possible with absolute confidence to extrapolate the results of vaccine studies in any single animal lentivirus system to other animal lentiviruses or to HIV, the data presented here certainly highlight the priority of ascertaining centralized immunogens on HIV vaccine efficacy in the context of higher animal models that include challenge studies which can inform on the true protective nature of the proposed immunogens.

## Materials and Methods

### Consensus Env and strains

Consensus gp90 Env protein sequence was determined by alignment of nucleotide (codon alignment) and amino acid sequences from naturally arisen EIAV isolates originated from an experimental infection (pony #567) [34], [42]–[44] in the Geneious Pro package of software (Biomatters, Ltd.). Alignments were hand edited where necessary, especially in the highly variable loop region, and ambiguities resolved through partner aligning of nucleotides and amino acids. Phylogenetic characterization of the consensus Env was were constructed by the neighbor-joining method of Jukes Cantor corrected distances with the optimality criterion set to distance as measured in PAUP [58] and implemented in the Geneious Pro 5.0.4 (Biomatters Ltd., NZ) package of software. Statistical significance of branchings and clustering was assessed by bootstrap resampling of 1000 pseudoreplicates on the complete data set; trees were rooted to the original infectious ancestral Env, EV0. The trees were edited for publication using FigTree Version 1.1.2. Resultant gp90 sequence was synthesized (GeneArt, Regensberg, Germany). The consensus gp90 was cloned into pathogenic and attenuated EIAV backbones using methods and restriction sites previously described [34], [47], [48]. Viral stocks were prepared as previously described [34], [48]. Viral stock titers were determined utilizing our infectious center assay (cell-based ELISA) in fetal equine kidney cells, described previously [46].

### Experimental infections with ConD9 and EVCon

All equine procedures were conducted in accordance with the recommendations in the Guide for the Care and Use of Laboratory Animals of the National Institutes of Health at the Gluck Equine Research Center of the University of Kentucky according to protocols approved by the University of Kentucky IACUC (#01058A2006). The animals were monitored daily and maintained as described previously [31], [32], [34], [43], [49]. Eight outbred, mixed-breed ponies were separated into two groups of four and experimentally inoculated intravenously with 10^3^ TCID_50_ of either chimeric strain ConD9 or EVCon. Rectal temperatures and clinical status were recorded daily. Platelet numbers were determined using the IDEXX VetAutoread Hematology Analyzer (IDEXX Laboratories Inc., Westbrook ME). Clinical EIA (fever) episodes were determined on the basis of rectal temperature and platelet count (rectal temperature >39°C; platelet number <100,000/µl of whole blood) in combination with the viremic presence of infectious plasma virus (≥10^5^) [20], [26], [43], [49], [59]. Samples of whole blood, serum, and plasma were collected weekly as well as daily during fever episodes. Plasma samples were stored at −80°C until used to determine plasma viral RNA level.

### Challenge strains

The challenge strain EV_MX_ quasispecies was produced by combining equivalent infectious titers (TCID_50_) of the variant challenge strains EV0, EV6, and EV13 [34]. Viral stocks were prepared as previously described [34], [48]. Viral stock titers were determined utilizing our infectious center assay (cell-based ELISA) in fetal equine kidney cells, described previously [46].

### Experimental subjects, vaccination, and challenge

Equine procedures were conducted at the Gluck Equine Research Center of the University of Kentucky according to protocols approved by the University of Kentucky IACUC. Thirty-six mixed age and gender outbred ponies, serognegative for EIAV, were utilized. Daily rectal temperatures and clinical status were recorded. CBC analysis of whole blood was performed using an IDEXX QBC Vet Autoreader. Hematocrit and platelet numbers were monitored weekly. The EIAV_D9_ stock was produced and vaccinations performed as described [31], [34]. Twenty-three vaccinated and six naïve ponies were challenged with 3×10^3^ TCID_50_ of EV_MX_. The ponies were monitored daily for clinical symptoms of EIA, and blood was drawn at regular intervals (weekly, daily if febrile) for assays of platelets, viral replication, and virus-specific immune responses. During the course of these experiments ponies that demonstrated severe disease-associated symptoms resulting in distress as outlined by the University of Kentucky IACUC were euthanized.

### Quantitative and Qualitative RT-PCR analysis of plasma virus RNA

Plasma samples from all subjects were analyzed for the levels of viral RNA per milliliter of plasma using a previously described quantitative real-time multiplex RT-PCR assay based on *gag*-specific amplification primers [60]. The standard RNA curve was linear in the range of 10^1^ molecules as a lower limit and 10^8^ molecules as an upper limit.

### Quantitative assays of cellular immune responses

Our *in vivo* method for assessing immune reactivity to specific peptides, described previously [61], was used to explore cellular immune responses. Forty-four 20-mer peptides, overlapping by 10 residues, spanning the EIAV_D9_ gp90 were generated (GenScript USA Inc., Piscataway, NJ). An additional 15 peptides, specific for the variable regions ConD9 gp90, were included in the pool for analysis of those vaccinates. Vaccinates were screened for gp90 specific cellular immune responses one week prior to day of challenge. A 2 mm skin biopsy was collected and homogenized and RNA extracted. IFNγ gene expression was determined by real-time PCR, as previously described [61]. Amplification efficiencies were determined using Linreg [62] and samples with amplification efficiencies above 99% were included for further analyses. Beta-glucuronidase (β-GUS) was used as housekeeping gene and the ΔΔCT method [63] was used to determine relative gene expression with saline injection site for each vaccinate used as the calibrator. Relative quantity (RQ) was calculated as 2^−ΔΔCT^.

### Quantitative and qualitative serological analyses

Serum IgG antibody reactivity to EIAV envelope glycoproteins was assayed quantitatively (end point titer) and qualitatively (avidity index, conformation ratio) using our standard concanavalin A (ConA) ELISA procedures as described previously [51]. Virus neutralizing activity to the historical reference strain EIAV_PV,_ and vaccine-specific virus stocks EVCon and EV_MX_, mediated by immune sera, was assessed in an indirect cell-ELISA based infectious center assay using a constant amount of infectious EIAV and sequential 2-fold dilutions of serum [46], [51].

### Statistical analyses

Significance of protection from disease was performed by survival curve analysis as implemented in GraphPad Prism version 6.0d (San Diego, CA). Significance of survival curves were determined utilizing One-way ANOVA with Bonferroni's multiple comparison's test as well as survival analysis of Kaplan Meier plots with Logrank test for trend.

### Ethics statement

All equine procedures were conducted in accordance with the recommendations in the Guide for the Care and Use of Laboratory Animals of the National Institutes of Health at the Gluck Equine Research Center of the University of Kentucky according to protocols approved by the University of Kentucky IACUC (#01058A2006).

## Supporting Information

S1 Fig **Day of challenge humoral immune responses in protected versus unprotected vaccinates.** Characterization of the quantitative and qualitative properties of induced EIAV envelope-specific humoral responses on the day of challenge were conducted in ConA serological ELISA assays of serum antibody (**A**) endpoint titer, (**B**) avidity, and (**C**) conformational dependence; (**D**) and 50% serum neutralization titer determinations, all as described in Materials and Methods. Animals in each vaccinate group were separated into one of two categories dependent on their protection or lack of protection from disease. (**A**) Mean serum antibody titers are presented as the log_10_ of the highest reciprocal dilution yielding reactivity two standard deviations above background. (**B**) Mean avidity index measurements are presented as percentages of the antibody-antigen complexes resistant to disruption with 8M urea. (**C**) Mean conformation dependence values are calculated as the ratio of serum antibody reactivity with native envelope compared to denatured envelope antigen. Conformation ratios greater than 1.0 indicate predominant antibody specificity for conformational determinants, while ratios less than 1.0 indicate predominant antibody specificity for linear envelope determinants. (**D**) The mean reciprocal dilutions of serum from vaccinated horses, which neutralized 50% of input EIAV_PV_, as measured in an infectious center assay. The grey dashed line denotes the cut off (≥25) value for valid 50% neutralization titers.
(TIF)
