# Supplementary figures and images for: Protective Efficacy of Centralized and Polyvalent Envelope Immunogens in an Attenuated Equine Lentivirus Vaccine
Source: PLoS Pathog. 2015 Jan 8;11(1):e1004610. doi: 10.1371/journal.ppat.1004610 (PMC4287611; doi:10.1371/journal.ppat.1004610)

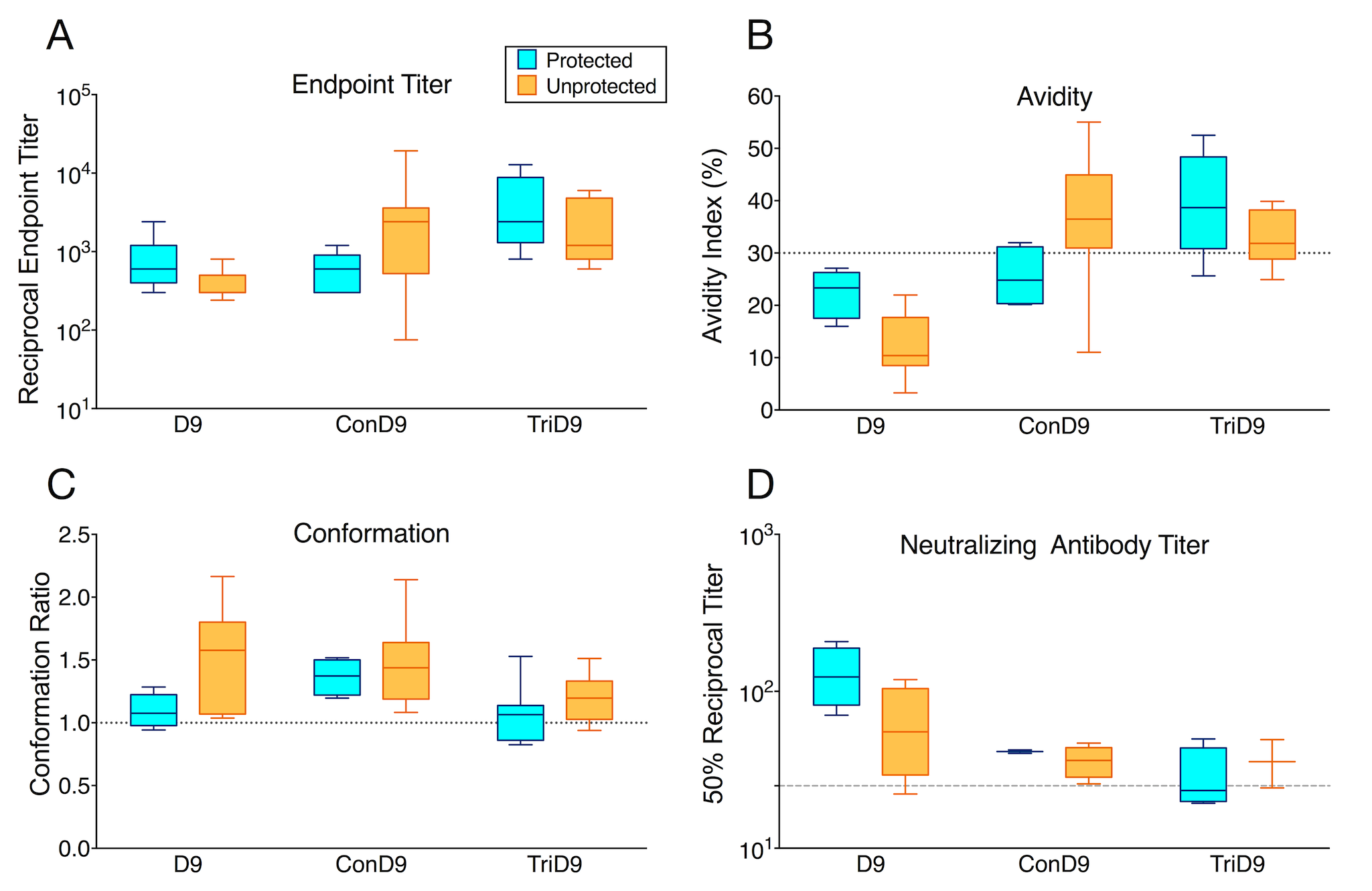

Supplement: S1 Fig — Day of challenge humoral immune responses in protected versus unprotected vaccinates. Characterization of the quantitative and qualitative properties of induced EIAV envelope-specific humoral responses on the day of challenge were conducted in ConA serological ELISA assays of serum antibody (A) endpoint titer, (B) avidity, and (C) conformational dependence; (D) and 50% serum neutralization titer determinations, all as described in Materials and Methods. Animals in each vaccinate group were separated into one of two categories dependent on their protection or lack of protection from disease. (A) Mean serum antibody titers are presented as the log10 of the highest reciprocal dilution yielding reactivity two standard deviations above background. (B) Mean avidity index measurements are presented as percentages of the antibody-antigen complexes resistant to disruption with 8M urea. (C) Mean conformation dependence values are calculated as the ratio of serum antibody reactivity with native envelope compared to denatured envelope antigen. Conformation ratios greater than 1.0 indicate predominant antibody specificity for conformational determinants, while ratios less than 1.0 indicate predominant antibody specificity for linear envelope determinants. (D) The mean reciprocal dilutions of serum from vaccinated horses, which neutralized 50% of input EIAVPV, as measured in an infectious center assay. The grey dashed line denotes the cut off (≥25) value for valid 50% neutralization titers. (TIF) [file ppat.1004610.s001.tif]
